# Serum fibroblast growth factor-23 concentration in dogs with myxomatous mitral valve disease

**DOI:** 10.1093/jvimsj/aalag265

**Published:** 2026-10-29

**Authors:** Naoki Iwasa, Mamu Shimizu, Tomoko Okamoto, Hiroto kosuga, Nayu Morikita, Mifumi Kawabe, Munetaka Iwata, Kazuhiro Watanabe, Yui Kobatake, Satoshi Takashima, Naohito Nishii

**Affiliations:** Hashima Animal Hospital, Gifu 501-6625, Japan; Joint Department of Veterinary Medicine, Faculty of Applied Biological Sciences, Gifu University, Gifu 501-1193, Japan; Hashima Animal Hospital, Gifu 501-6625, Japan; Joint Department of Veterinary Medicine, Faculty of Applied Biological Sciences, Gifu University, Gifu 501-1193, Japan; Hashima Animal Hospital, Gifu 501-6625, Japan; Hashima Animal Hospital, Gifu 501-6625, Japan; Hashima Animal Hospital, Gifu 501-6625, Japan; Hashima Animal Hospital, Gifu 501-6625, Japan; Gifu University Animal Medical Center, Gifu 501-1193, Japan; Hashima Animal Hospital, Gifu 501-6625, Japan; Orchid Animal Oncology and Orthopedic Center, Tokyo 120-0035, Japan; Hashima Animal Hospital, Gifu 501-6625, Japan; Joint Department of Veterinary Medicine, Faculty of Applied Biological Sciences, Gifu University, Gifu 501-1193, Japan; Joint Department of Veterinary Medicine, Faculty of Applied Biological Sciences, Gifu University, Gifu 501-1193, Japan; Joint Department of Veterinary Medicine, Faculty of Applied Biological Sciences, Gifu University, Gifu 501-1193, Japan; Joint Department of Veterinary Medicine, Faculty of Applied Biological Sciences, Gifu University, Gifu 501-1193, Japan

**Keywords:** ACVIM stage, biomarkers, dogs, fibroblast growth factor-23, left atrial enlargement, myxomatous mitral valve disease

## Abstract

**Background:**

Circulating fibroblast growth factor-23 (FGF-23) is primarily a kidney-related biomarker, but also may be influenced by cardiovascular remodeling and hemodynamic load.

**Hypothesis/Objectives:**

Verify whether serum FGF-23 concentration increases with myxomatous mitral valve disease (MMVD) severity.

**Animals:**

Forty-two client-owned dogs with MMVD without overt kidney disease.

**Methods:**

Retrospective cross-sectional study to evaluate serum FGF-23, N-terminal pro-B-type natriuretic peptide (NT-proBNP), atrial natriuretic peptide (ANP), blood urea nitrogen (BUN), creatinine (Cr), and symmetric dimethylarginine (SDMA) concentrations. Imaging variables included vertebral heart score (VHS), vertebral left atrial size (VLAS), left atrial-to-aortic root ratio (LA/Ao), normalized left-ventricular internal diameter in diastole (LVIDDN), and diastolic transmitral E-wave velocity (E-vel). Differences among American College of Veterinary Internal Medicine stages were analyzed using the Kruskal–Wallis test with Bonferroni-adjusted pairwise comparisons. Correlations were analyzed using Spearman’s correlation. Multivariable linear regression analysis was performed to identify factors associated with serum FGF-23 concentration.

**Results:**

Dogs with ACVIM stages C and D had higher serum FGF-23 concentrations than those with stages B1 and B2 (*P* < .05). Serum FGF-23 concentrations were positively correlated with VLAS (*rs* = 0.500, *P* < .01), ANP (*rs* = 0.455, *P* < .01), LA/Ao (*rs* = 0.384, *P* < .05), E-vel (*rs* = 0.372, *P* < .05), and NT-proBNP (*rs* = 0.366, *P* < .05). ACVIM stage was significantly associated with serum FGF-23 concentration after adjustment for serum Cr concentration and loop diuretic use.

**Conclusions and clinical importance:**

The potential influence of MMVD should be considered when interpreting increased circulating FGF-23 concentrations in dogs with MMVD.

## Introduction

Fibroblast growth factor-23 (FGF-23) is a phosphaturic hormone secreted mainly by osteocytes and osteoblasts.^1^^,^^2^ It acts primarily on the kidneys, where it suppresses sodium-phosphate cotransporters in the proximal tubules and decreases calcitriol synthesis, thereby promoting urinary phosphate excretion and contributing to phosphate and vitamin D homeostasis.^1–3^ In chronic kidney disease (CKD), impaired renal phosphate excretion leads to phosphate retention, which stimulates FGF-23 secretion. Accordingly, circulating FGF-23 concentration increases as renal function declines and is closely associated with kidney disease.^1–3^ In dogs with CKD, circulating FGF-23 concentration correlates with disease severity,^4^ has prognostic value,^5^ and is associated with serum phosphate concentration.^6^ Collectively, these findings support the role of circulating FGF-23 as a kidney disease-related biomarker in veterinary medicine.^4–6^

In human medicine, circulating FGF-23 concentration is associated not only with kidney disease^7^ but also with cardiovascular disease.^8^ Increased circulating FGF-23 has been linked to cardiac remodeling, including left ventricular hypertrophy and left atrial enlargement, and also may reflect cardiovascular risk.^8–13^ Proposed mechanisms include direct hypertrophic signaling in cardiomyocytes,^11–13^ effects on renin angiotensin aldosterone system (RAAS)-related pathways supported by the positive association between serum aldosterone concentration and circulating FGF-23 concentration across multiple species, including dogs,^14^ promotion of interactions between cardiomyocytes and cardiac fibroblasts,^15^ and kidney injury-related mechanisms associated with renal ischemia or decreased renal perfusion.^16^ These observations support the possibility that cardiac disease and its hemodynamic consequences may influence circulating FGF-23 concentration independently of primary renal dysfunction.^8–16^

Myxomatous mitral valve disease (MMVD) is the most common acquired cardiac disease in small breed dogs.^17^^,^^18^ It is characterized by chronic volume overload, progressive left atrial enlargement, and left ventricular eccentric remodeling.^17^^,^^18^ If MMVD influences circulating FGF-23 concentration in dogs, this effect should be considered when interpreting circulating FGF-23 as a renal biomarker. At the same time, circulating FGF-23 may have potential utility as an adjunctive biomarker associated with cardiac remodeling and hemodynamic load in dogs with MMVD. We hypothesized that serum FGF-23 concentration would increase with MMVD severity and be associated with indices of left atrial enlargement and volume load. Accordingly, we measured serum FGF-23 concentrations in dogs across different severities of MMVD, examined their associations with echocardiographic indices, and assessed whether MMVD severity itself was associated with serum FGF-23 concentration in dogs without overt kidney disease.

## Materials and methods

Our retrospective cross-sectional study included client-owned dogs diagnosed with MMVD at Hashima Animal Hospital between June 2022 and September 2025. Dogs were classified as American College of Veterinary Internal Medicine (ACVIM) stage B1, B2, C, or D according to consensus criteria.^18^ Dogs were excluded if they had concurrent systemic disease, incomplete data, or evidence of renal disease. Renal exclusion criteria were serum creatinine (Cr) ≥1.4 mg/dL, symmetric dimethylarginine (SDMA) ≥18 μg/dL, relevant renal ultrasonographic abnormalities, previous acute kidney injury, or congenital kidney or urinary tract anomalies.^19^ Dogs with previous mitral valve repair were excluded because surgery alters cardiac structure and biomarkers.^20^^,^^21^

Signalment, body weight, clinical signs, medication history, and cardiac and renal variables were extracted from medical records. Biomarkers consisted of serum FGF-23, N-terminal pro-B-type natriuretic peptide (NT-proBNP), blood urea nitrogen (BUN), Cr, inorganic phosphorus, calcium, SDMA, cystatin C (Cys-C), and aprotinin plasma atrial natriuretic peptide (ANP). Imaging variables consisted of vertebral heart score (VHS), vertebral left atrial size (VLAS), interventricular septal thickness (IVSd), left ventricular posterior wall thickness (LVPWd), left atrial-to-aortic root ratio (LA/Ao), normalized left ventricular internal diameter in diastole (LVIDDN), and early diastolic transmitral E-wave velocity (E-vel). Cardiac biomarkers were not used for ACVIM staging. The VHS and VLAS were measured as described previously.^22–24^ All echocardiograms were performed by one veterinarian; LA/Ao and LVIDDN were determined using established methods.^25^^,^^26^ Routine samples were analyzed by commercial laboratories. Cystatin C was measured by a latex immunoturbidimetric assay validated for use in dogs^27^; FGF-23 by an ELISA validated for use in dogs,^28^^,^^29^ with results > 4000 pg/mL right-censored; and ANP by chemiluminescent enzyme immunoassay.^30^

Continuous variables are reported as median (range). Differences among ACVIM stages were assessed using Kruskal–Wallis tests followed, when significant, by pairwise Mann–Whitney U tests with Bonferroni correction. Associations between FGF-23 and other variables were assessed using Spearman rank correlation. A prespecified multivariable linear regression model evaluated the association between ACVIM stage and log10-transformed FGF-23 after adjustment for serum Cr concentration and loop diuretic use; stage B1 was the reference. Analyses were 2-sided, with *P* < .05 considered significant, and were performed using R version 4.1.1 with EZR.^31^^,^^32^ Full procedural, assay, and statistical details are provided in the Appendix.

The ethics committee of Gifu University approved the study (E24010), waived written owner consent for the use of anonymized routine clinical data, and permitted an opt-out procedure.

## Results

### Characteristics of included dogs

From June 2022 to September 2025, 82 dogs were diagnosed with MMVD. One dog was excluded because of hyperadrenocorticism. Of the remaining 81 dogs with MMVD, 16 had incomplete data, 17 had Cr ≥ 1.4 mg/dL or SDMA ≥18 μg/dL or both, 4 had renal abnormalities on ultrasonography, and 2 had previous mitral valve repair surgery. All of these dogs were excluded from the study. Ultimately, 42 dogs were included in the analysis (Figure 1). Among the enrolled dogs, 7 were intact males, 18 were neutered males, 1 was an intact female, and 16 were spayed females. The various breeds included 13 Chihuahuas, 9 Toy Poodles, 6 mixed breeds, 5 Pomeranians, 3 Miniature Schnauzers, 2 Shih Tzus, 1 Papillon, 1 Yorkshire Terrier, 1 Maltese, and 1 Shiba Inu. Details on the characteristics of the enrolled dogs and their MMVD stages are presented in Table 1. Based on the ACVIM consensus guidelines for MMVD, 23 dogs were classified as stage B1, 8 were stage B2, 5 were stage C, and 6 were stage D. Dogs in stage B1 were asymptomatic and were not receiving treatment for MMVD. Among the 8 dogs classified as stage B2, 3 were exhibiting cough, and 4 were being treated with pimobendan at the time of sampling and the remaining 4 dogs received pimobendan after diagnosis. In the dogs classified as stage C, clinical signs observed included cough (2/5), exercise intolerance (4/5), dyspnea (4/5), tachypnea or labored respiration with pulmonary edema (4/5), history of cardiac pulmonary edema (1/5), and cyanosis (2/5). At the time of sampling, these dogs were being treated with angiotensin-converting enzyme inhibitors (2/5), amlodipine (1/5), pimobendan (3/5), or furosemide (1/5) or some combination of these. The remaining 4 dogs were not receiving furosemide at the time of sampling but they were judged to have pulmonary edema at the clinical assessment used for staging and were started on furosemide after that assessment. The 6 dogs categorized in stage D exhibited clinical signs such as cough (5/6), exercise intolerance (2/6), dyspnea (5/6), tachypnea or labored respiration because of pulmonary edema (1/6), history of pulmonary edema (6/6), and cyanosis (2/6). These dogs were treated with spironolactone (6/6), sildenafil (1/6), amlodipine (4/6), pimobendan (6/6), furosemide (6/6), or some combination of these.

**Figure 1:**
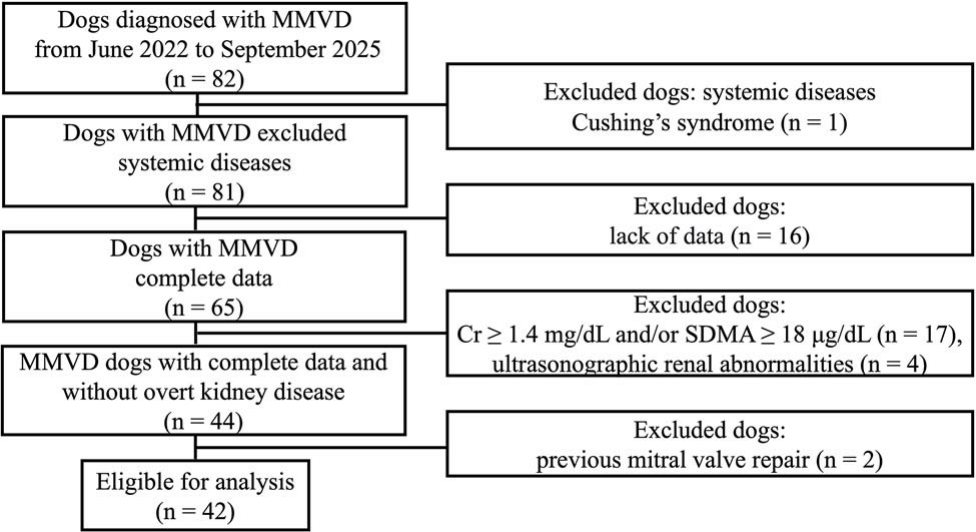
Flow diagram illustrating the enrollment of dogs with myxomatous mitral valve disease.

**Table 1:** Characteristics of dogs by the ACVIM stages.

**Parameters**	**All (*n* = 42)**	**Stage B1 (*n* = 23)**	**Stage B2 (*n* = 8)**	**Stage C (*n* = 5)**	**Stage D (*n* = 6)**
**Age (year)**	12 (5.0-15.0)	12.0 (5.0-15.0)	12.0 (5.0-15.0)	12.0 (11.0-13.0)	13.0 (9.0-15.0)
**Body weight (kg)**	4.32 (2.4-11.7)	4.7 (2.4-11.7)	4.6 (2.5-7.0)	3.7 (3.3-9.1)	3.3 (3.1-3.6)
**Sex(M/F)**	25/17	13/10	6/2	2/3	4/2
**VHS**	10.9 (9.0-13.0)	10.5 (9.0-11.6)	11.0 (10.6-11.5)	12.3 (10.7-13.0)^*^	12.1 (11.1-12.5)^*^
**VLAS**	2.5 (1.7-3.5)	2.2 (1.7-2.7)	2.5 (2.0-3.0)	3.0 (2.8-3.5)^**^	2.9 (2.6-3.2)^**^
**IVSd (mm)**	6.8 (3.8-11.6)	8.0 (3.8-11.6)	5.7 (4.6-10.9)	6.5 (4.1-6.9)	6.6 (4.8-8.8)
**LVPWd (mm)**	6.0 (3.7-10.9)	6.3 (4.8-10.0)	5.5 (3.7-6.6)	5.5 (4.3-7.8)	5.3 (4.8-10.9)
**LA/Ao**	1.56 (1.03-2.75)	1.30 (1.03-1.66)	1.84 (1.62-2.44)^**^	1.96 (1.56-2.75)^**^	1.89 (1.65-2.21)^**^
**LVIDDN (cm/kg0.294)**	1.74 (1.2-2.5)	1.56 (1.20-2.12)	1.92 (1.71-2.42)^*^	2.10 (1.72-2.46)^*^	1.91 (1.25-1.97)
**E-vel (m/s)**	0.91 (0.53-1.50)	0.80 (0.53-1.44)	1.02 (0.9-1.11)	1.16 (0.97-1.23)^*^	1.02 (0.81-1.50)^**^
**NT-proBNP (pmol/L)**	1082 (250-5674)	657 (250-2113)	1194 (909-2591)	2082 (746-3656)^*^	3137 (779-5674)^*^
**ANP (pg/mL)**	119.2 (55.6-784.4)	106.0 (55.6-250.3)	107.3 (61.8-301.2)	272.8 (120.0-784.4)^**^	263.7 (165.6-737.0)^**^
**BUN (mg/dL)**	20.7 (10.1-49.7)	19.6 (10.1-47.2)	21.3 (16.5-37.5)	26.1 (12.4-31.7)	26.4 (14.1-49.7)
**Cr (mg/dL)**	0.8 (0.45-1.36)	0.77 (0.45-1.36)	0.83 (0.45-1.27)	0.69 (0.56-1.34)	0.82 (0.65-1.09)
**Inorganic phosphorus (mg/dL)**	3.4 (1.3-7.8)	3.2 (2.1-5.0)	3.1 (1.3-4.6)	4.3 (3.4-5.8)	3.7 (2.7-7.8)
**Calcium (mg/dL)**	10.2 (7.0-12.2)	10.1 (9.0-11.7)	10.5 (8.6-11.7)	10.1 (9.2-11.3)	10.6 (7.0-12.2)
**SDMA (μg/dL)**	11 (7-17)	10 (7-17)	10 (8-15)	12 (9-14)	12 (7-15)
**Cys-C (mg/L)**	0.32 (0.16-0.67)	0.29 (0.16-0.53)	0.31 (0.22-0.50)	0.32 (0.27-0.39)	0.46 (0.37-0.67)
**FGF-23 (pg/mL)**	354 (25-1654)	276 (25-941)	225 (35-708)	865 (681-1169)^*,^^†^	848 (428-1654)^*,^^†^

Abbreviations: ANP = atrial natriuretic peptide; BUN = blood urea nitrogen; Cr = creatinine; Cys-C = cystatin C; E-vel = early diastolic transmitral E-wave velocity; FGF-23 = fibroblast growth factor-23; IVSd = end-diastolic intraventricular septum; LA/Ao = left atrial-to-aortic ratio; LVIDDN = left ventricular end-diastolic internal diameter; LVPWd = end-diastolic left ventricular posterior wall; NT-proBNP = N-terminal pro-B-type natriuretic peptide; SDMA = symmetric dimethylarginine; VHS = vertebral heart score; VLAS = vertebral left atrial size.

^*^Significantly different from stage B1 (*P* < .05).^**^Significantly different from stage B1 (*P* < .01).

^†^Significantly different from stage B2 (*P* < .05).

Regarding medication dosages, dogs in stage C treated with pimobendan received a median dosage of 0.6 mg/kg/day (0.5-0.8 mg/kg/day), whereas 1 dog received furosemide at 3 mg/kg/day. Dogs in stage D received pimobendan at 0.8 mg/kg/day (0.6-1.5 mg/kg/day), furosemide at 4.4 mg/kg/day (3.6-6.4 mg/kg/day), or spironolactone at 4.2 mg/kg/day (3.8-4.8 mg/kg/day). Dogs were classified as ACVIM stage D when they had persistent or recurrent signs of congestive heart failure, including repeated episodes of pulmonary edema, despite intensification of standard stage C treatment. Stage D classification was based on overall clinical assessment and was not determined solely by the furosemide dosage threshold. In dogs classified as stage D, diuretic treatment was further intensified after the examination. Also, some dogs in ACVIM stages C and D received pimobendan at dosages exceeding the currently recommended dosage; these higher dosages reflected clinician-directed off-label use in dogs with advanced or refractory disease.

### Differences by ACVIM stages

The box-and-whisker plot of serum FGF-23 concentrations in dogs at different MMVD stages is shown in Figure 2. Dogs in stages C and D had higher serum FGF-23 concentrations than those in stages B1 and B2 (Bonferroni-adjusted *P* values: B1 vs C, *P* = .02; B2 vs C, *P* = .02; B1 vs D, *P* = .02; B2 vs D, *P* = .02). Compared with those in stage B1, VHS, VLAS, E-vel, NT-proBNP, and ANP were significantly higher in stages C and D; LA/Ao was higher in stages B2, C, and D; and LVIDDN was higher in stages B2 and C (Table 1). However, no significant differences among stages were observed for age, body weight, sex distribution, IVSd, LVPWd, BUN, Cr, inorganic phosphorus, calcium, SDMA, or Cys-C.

**Figure 2:**
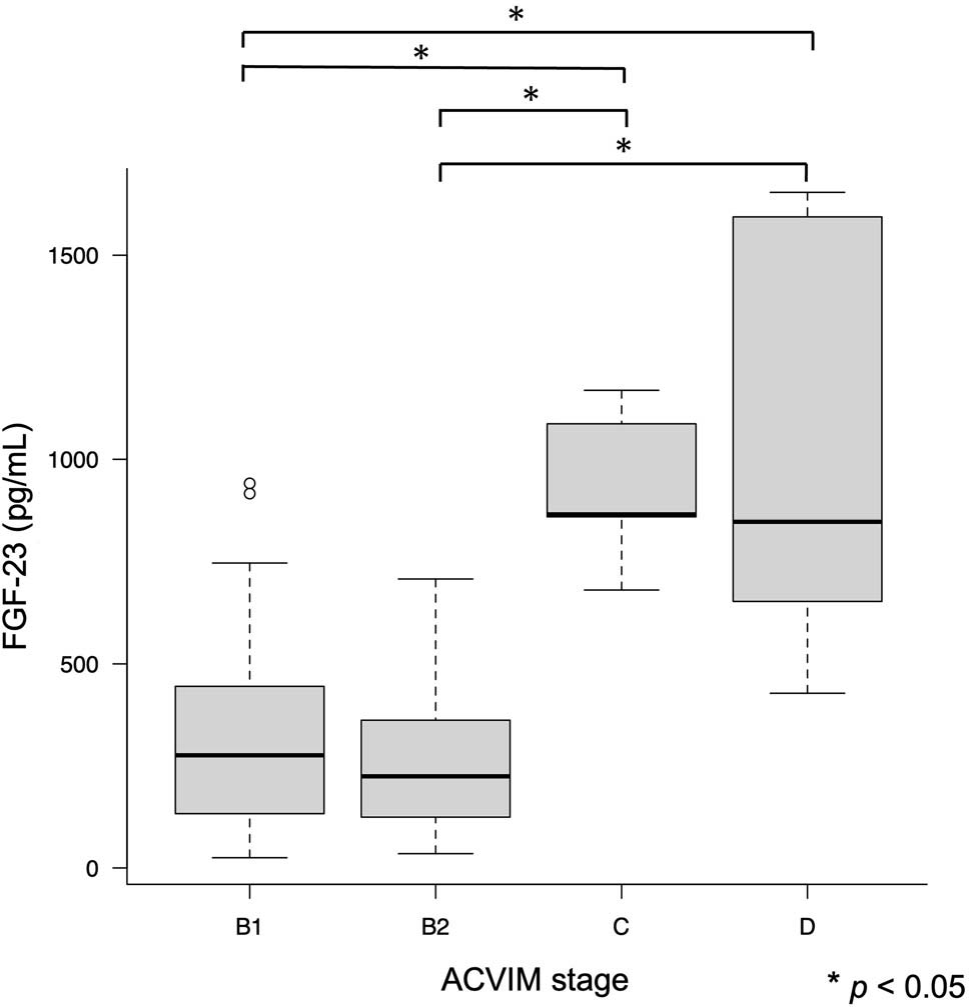
Box-and-whisker plot of serum FGF-23 concentrations in dogs at different stages of myxomatous mitral valve disease. Twenty-three dogs were stage B1, 8 dogs were stage B2, 5 dogs were stage C, and 6 dogs were stage D. The dogs in stages C and D had higher serum FGF-23 concentrations than those in stages B1 and B2 (all *P* < .05).

### Correlations between serum FGF-23 concentration and other variables

Correlations between serum FGF-23 concentration and other variables are shown in Table 2, and corresponding scatterplots are provided in Figure S1. In particular, serum FGF-23 concentrations were positively correlated with VLAS (*rs* = 0.500, *P* < .01), ANP (*rs* = 0.455, *P* < .01), LA/Ao (*rs* = 0.384, *P* < .05), E-vel (*rs* = 0.372, *P* < .05), and NT-proBNP (*rs* = 0.366, *P* < .05).

**Table 2:** Correlations between serum FGF-23 concentration and other variables.

**Variables**	***n***	***rs***	***P***
**VHS**	42	0.192	.22
**VLAS**	42	0.500	<.01
**IVSd (mm)**	42	−0.214	.17
**LVPWd (mm)**	42	−0.203	.20
**LA/Ao**	42	0.384	<.05
**LVIDDN (cm/kg^0.294^)**	42	0.215	.17
**E-vel (m/s)**	42	0.372	<.05
**NT-proBNP (pmol/L)**	42	0.366	<.05
**ANP (pg/mL)**	42	0.455	<.01
**BUN (mg/dL)**	42	0.118	.46
**Cr (mg/dL)**	42	0.051	.75
**Inorganic phosphorus (mg/dL)**	42	0.300	.06
**Calcium (mg/dL)**	42	−0.093	.56
**SDMA (µg/dL)**	42	0.028	.86
**Cys-C (mg/L)**	42	0.281	.07

Abbreviations: ANP = atrial natriuretic peptide; BUN = blood urea nitrogen; Cr = creatinine; Cys-C = cystatin C; E-vel = early diastolic transmitral E-wave velocity; FGF-23 = fibroblast growth factor-23; IVSd = end-diastolic intraventricular septum; LA/Ao = left atrial-to-aortic ratio; LVIDDN = left ventricular end-diastolic internal diameter; LVPWd = end-diastolic left ventricular posterior wall; NT-proBNP = N-terminal pro-B-type natriuretic peptide; SDMA = symmetric dimethylarginine; VHS = vertebral heart score; VLAS = vertebral left atrial size.

### Multivariable linear regression for serum FGF-23 concentration

After log transformation and adjustment for serum Cr concentration and loop diuretic use at the time of sampling, ACVIM stages C and D were significantly associated with serum FGF-23 concentration compared with stage B1, whereas stage B2, serum Cr concentration, and loop diuretic use were not significantly associated (Table 3). Residual normality of the final model was considered acceptable based on the histogram, normal Q–Q plot, and Shapiro–Wilk test (W = 0.969, *P* = .3). The model explained 39% of the variance in serum FGF-23 concentration (*R*^2^ = 0.39, adjusted *R*^2^ = 0.31).

**Table 3:** Multivariable linear regression of factors associated with log10-transformed serum FGF-23.

**Variable**	**β**	**95% CI lower**	**95% CI upper**	**SE**	***P***
**Intercept**	2.3195	1.8513	2.7877	0.2308	<.01
**ACVIM stage B2**	−0.0527	−0.3631	0.2576	0.1530	.73
**ACVIM stage C**	0.6297	0.2389	1.0205	0.1927	<.01
**ACVIM stage D**	0.6785	0.0752	1.2818	0.2975	<.05
**Creatinine**	0.0389	−0.5114	0.5892	0.2713	.89
**Loop diuretic use**	−0.0956	−0.6949	0.5037	0.2955	.75

## Discussion

In our study involving 42 dogs with MMVD but no overt kidney disease, circulating FGF-23 was higher in dogs with clinically advanced disease (ACVIM stages C and D) than in those with preclinical disease. This association remained significant after adjustment for serum Cr concentration and loop diuretic use, whereas stage B2 did not differ from stage B1. Circulating FGF-23 was also positively correlated with VLAS, LA/Ao, ANP, E-vel, and NT-proBNP, although these correlations were weak. Together, these findings suggest that serum FGF-23 concentration increases with indices related to cardiac remodeling and higher left-sided filling pressures, although other explanations for these observations are possible. These findings are consistent with studies in humans reporting an association between circulating FGF-23 concentration and cardiac disease severity.^8^ Furthermore, the association between serum FGF-23 concentration and left atrial size observed in our study also has been reported in humans.^9^^,^^10^ We also found weak associations between serum FGF-23 concentration and indices of left atrial enlargement and volume load. Progression of MMVD results in volume overload and left atrial stretch^17^^,^^18^^,^^25^^,^^33^ alongside neurohormonal activation,^34^ including RAAS.^35^^,^^36^ Prior studies in humans indicate that circulating FGF-23 can both indirectly and directly interact with the RAAS.^11^^,^^12^ Furthermore, studies in dogs, cats, and mice have found a link between circulating FGF-23 concentration and serum aldosterone concentration, an index of RAAS activity.^14^ Because RAAS components were not evaluated in our study, the underlying mechanisms in dogs could not be verified.

Serum FGF-23 concentration increases with declining renal function^4^^,^^5^ and is correlated with serum Cr concentration.^4^^,^^28^ Therefore, to minimize the inclusion of dogs with overt renal dysfunction, we deliberately selected dogs with Cr < 1.4 mg/dL, SDMA < 18 μg/dL, and no structural renal abnormalities on imaging. However, mild pre-renal dysfunction could not be completely excluded. In dogs with advanced MMVD, decreased forward cardiac output and diuretic treatment may have contributed to renal hypoperfusion even in the absence of overt primary renal disease.^37^ Nevertheless, the potential influence of renal function on serum FGF-23 concentration was examined in our study using multivariable linear regression analysis including serum Cr concentration and loop diuretic use as explanatory variables. In this model, serum FGF-23 concentration was not significantly associated with either serum Cr concentration or loop diuretic use. Nevertheless, the lack of statistical significance does not exclude the potential effects of pre-renal factors or subclinical renal impairment undetectable by serum Cr or SDMA measurements. Taken together, these data indicate that increased serum FGF-23 concentration in dogs with MMVD may occur through mechanisms that are at least partially independent of overt primary renal dysfunction and that are associated with disease severity and cardiac-related factors.

Previous studies in humans have suggested that diuretics can affect circulating FGF-23 concentrations.^8^^,^^38^ Although diuretic use was not significantly associated with serum FGF-23 concentrations in the multivariable analysis, many dogs with stage C and all dogs with stage D had already received medications at the time of blood sample collection. Therefore, the potential influence of medications on circulating FGF-23 concentrations cannot be completely excluded.

Serum FGF-23 concentration was not associated with IVSd or LVPWd in our study. In human medicine, circulating FGF-23 concentration can directly or indirectly influence left ventricular myocardial wall thickening.^10–13^ However, MMVD in dogs is a volume-overload disease characterized mainly by left ventricular eccentric hypertrophy and progressive left atrial enlargement.^17^^,^^18^^,^^24^^,^^25^ Therefore, the disparity in the correlation between serum FGF-23 concentrations and myocardial wall thickness parameters may be attributed to differences in cardiac load and the underlying pathophysiological differences between cardiac disease in dogs and humans. In dogs with MMVD, circulating FGF-23 may be interpreted not as a biomarker of myocardial wall thickening, but as an adjunctive marker associated with left atrial enlargement and volume overload.

Our study had some limitations. First, small sample size limited statistical power and the precision of the estimates obtained. In addition, the group sizes were unequal across ACVIM stages, particularly in stages B2, C, and D, which may have affected the precision of between-group comparisons. Second, an age-matched healthy control group was not included. Therefore, the absolute magnitude of increase in FGF-23 concentration and the true reference range in clinically healthy dogs of similar age could not be fully evaluated. Third, feeding schedules and dietary phosphate intake were not standardized, which may have influenced serum FGF-23 concentrations. However, the absence of significant differences in serum phosphorus concentration among ACVIM stages does not exclude such an effect. Because serum phosphorus concentration is influenced by several regulatory factors, single-point measurements may not adequately reflect dietary phosphate exposure. Therefore, stricter control of dietary phosphate intake might have allowed a more appropriate assessment of serum FGF-23 concentration independent of dietary effects. Fourth, urinalysis-based markers (eg, tubular injury or proteinuria indices) were not uniformly available, indicating the possible inclusion of early but undetected kidney disease. In addition, RAAS-related variables and arterial blood pressure were not evaluated. Inclusion of these variables might have provided additional insight into the mechanisms associated with increased FGF-23 concentration, but interpretation of RAAS activity would have remained complicated in dogs receiving diuretics.

In conclusion, serum FGF-23 concentration increased with MMVD severity and was weakly correlated with indices of left atrial size and load in dogs without overt kidney disease. Assessments of circulating FGF-23 concentration in dogs with MMVD must take into account the potential influence of MMVD itself on increased FGF-23 concentrations.

## Supplementary Material

aalag265s1

## Off-label antimicrobial declaration

Authors declare no off-label use of antimicrobials.

## Institutional animal care and use committee or other approval declaration

Approved by the ethics committee for animal clinical research of Gifu University (E24010).

## Appendix

### Materials and methods

#### Case selection

Our retrospective cross-sectional study enrolled client-owned dogs diagnosed with MMVD at Hashima Animal Hospital between June 2022 and September 2025. Dogs with MMVD that were included in the study were classified into MMVD stages (B1, B2, C, or D) based on the ACVIM consensus guidelines.^18^ The distinction between stages B1 and B2 was based on the presence or absence of structural cardiac remodeling assessed using echocardiographic and thoracic radiographic findings in addition to clinical murmur status. Dogs that did not fulfill the criteria for stage B2 were classified as stage B1. Data extracted from medical records included medication history, clinical signs, body weight, age, sex, breed, and serum biomarkers. The biomarkers analyzed in serum included FGF-23, N-terminal pro-B-type natriuretic peptide (NT-proBNP), atrial natriuretic peptide (ANP; measured in aprotinin plasma), blood urea nitrogen (BUN), Cr, inorganic phosphorus, calcium, SDMA, and cystatin C (Cys-C). The imaging variables evaluated in the analysis included vertebral heart score (VHS), vertebral left atrial size (VLAS), interventricular septal thickness (IVSd), left ventricular posterior wall thickness (LVPWd), left atrial-to-aortic root ratio (LA/Ao), normalized left-ventricular internal diameter in diastole (LVIDDN), and early diastolic transmitral E-wave velocity (E-vel). Although several of these biomarkers are not in the standard ACVIM assessment, they are routinely assessed at our institution to obtain a more comprehensive evaluation of cardiac and renal function. Biomarkers such as ANP and NT-proBNP were not used for ACVIM stage classification and were analyzed only as study variables. Dogs that met any of the following criteria were excluded: (1) concurrent systemic diseases; (2) missing data; and (3) indicators of renal disease. Renal exclusions, applied to minimize confounding associated with impaired renal function, were based on International Renal Interest Society guidelines^19^ and included serum creatinine (Cr) concentration ≥ 1.4 mg/dL or symmetric dimethylarginine (SDMA) concentration ≥ 18 μg/dL or both, relevant ultrasonographic renal abnormalities (eg, multiple cortical cysts, irregular renal margins, or markedly decreased renal size), a previous diagnosis of acute kidney injury or congenital kidney and urinary tract anomalies. Finally, dogs that had previously undergone mitral valve repair surgery were excluded because of the potential changes induced by surgical correction on cardiac structure, load conditions, and circulating biomarker concentrations.^20^^,^^21^ The study was approved by the ethics committee for animal clinical research of Gifu University (Approval No. E24010). Because it was a retrospective study using anonymized clinical data obtained during routine clinical practice, and no additional blood sampling, diagnostic testing, or intervention was performed for the purpose of the study, the requirement for written informed owner consent was waived by the ethics committee. Owners were given the opportunity to opt out through a notice posted on the hospital bulletin board.

#### Chest radiography

VHS^18^^,^^22^^,^^23^ and VLAS^18^^,^^24^ were measured using right lateral thoracic radiographs, as described previously.

#### Echocardiography

Two-dimensional, M-mode, and Doppler echocardiography were performed by a single veterinarian using an ultrasound system (Noblus, FUJIFILM Healthcare, Tokyo, Japan) with a 2.0-9.0-MHz sector transducer (S-31, FUJIFILM Healthcare). Dogs were positioned in right lateral recumbency to obtain right parasternal views and in left lateral recumbency for left apical Doppler recordings. The LA/Ao was measured from the right parasternal short-axis view in the first frame after aortic valve closure, following method a previously described.^25^ The LVIDDN was calculated as LVIDd (cm)/body weight (kg)^0.294^,^18^^,^^26^ with the LVIDd obtained from M-mode. The E-vel was recorded using pulsed-wave Doppler from the left apical 4-chamber view.

#### Blood biochemistry

Blood samples were collected as part of routine clinical evaluation and processed according to the standard clinical protocol at our hospital. No additional blood samples were collected specifically for the study, and no stored residual samples were used for additional research-specific analyses. Serum and plasma samples were analyzed by commercial laboratories using routine clinical procedures. Serum NT-proBNP and SDMA were analyzed by IDEXX Laboratories (Tokyo, Japan). Serum BUN, Cr, inorganic phosphorus, calcium, Cys-C, FGF-23, and aprotinin plasma ANP were analyzed by Animal Medical Technology (Aichi, Japan). Cys-C concentrations were determined using a latex immunoturbidimetric assay designed for humans and previously validated for use in dogs.^27^ The FGF-23 concentrations were measured in serum using an FGF-23 enzyme-linked immunosorbent assay (ELISA) Kit (Kainos Laboratories, Tokyo, Japan), which had been previously validated for use in dogs with intra- and inter-assay coefficients of variation < 15%.^28^ The assay had a lower limit of detection of 3 pg/mL and an analytical quantification range of 3-800 pg/mL.^4^^,^^28^ To extend the measurable range up to 4000 pg/mL, samples were diluted accordingly. Then, results > 4000 pg/mL were right-censored and reported as > 4000 pg/mL.^28^^,^^29^ Plasma ANP was measured by chemiluminescent enzyme immunoassay used in prior veterinary validation studies.^30^

#### Statistical analysis

Continuous variables are expressed as median (range). Statistical analyses were performed using R (version 4.1.1; R Foundation for Statistical Computing, Vienna, Austria) with EZR (Saitama Medical Center, Jichi Medical University).^31^^,^^32^ Differences in all valuables among ACVIM stages were analyzed using the Kruskal–Wallis test. When the overall test was significant, pairwise Mann–Whitney U tests with Bonferroni adjustment across all stage pairs (*n* = 6) were performed to control for a Type I error because of multiple comparisons resulting in a more stringent significance threshold. Associations between serum FGF-23 concentration and other variables were assessed using Spearman’s rank correlation coefficient (rs). Finally, to identify factors associated with serum FGF-23 concentration, a multivariable linear regression model was fitted. To improve model fit and better satisfy the assumptions of linear regression, serum FGF-23 concentration was log10-transformed before analysis. Residual normality was assessed using histograms, normal Q–Q plots, and the Shapiro–Wilk test. Multivariable linear regression then was performed with log10-transformed serum FGF-23 concentration as the dependent variable. Independent variables included ACVIM stage, which was treated as a categorical variable with stage B1 as the reference category (indicator variables for stages B2, C, and D), serum Cr concentration (mg/dL; continuous), and loop diuretic use (yes/no). Variables included in the multivariable analysis were selected a priori based on clinical and biological plausibility. The model was constructed to assess whether the association between ACVIM stage and serum FGF-23 concentration remained after adjustment for serum Cr concentration and loop diuretic use. Analyses were 2-sided, and *P* < .05 was considered statistically significant.

## Abbreviations

ACVIM: American College of Veterinary Internal Medicine
ANP: atrial natriuretic peptide
BUN: blood urea nitrogen
Cr: creatinine
Cys-C: cystatin C
E-vel: early diastolic transmitral E-wave velocity
FGF-23: fibroblast growth factor-23
IVSd: end-diastolic intraventricular septum
LA/Ao: left atrial-to-aortic ratio
LVIDDN: left ventricular end-diastolic internal diameter
LVPWd: end-diastolic left ventricular posterior wall
MMVD: myxomatous mitral valve disease
NT-proBNP: N-terminal pro-B-type natriuretic peptide
SDMA: symmetric dimethylarginine
VHS: vertebral heart score
VLAS: vertebral left atrial size
